# The regulation and potential role of interleukin-32 in tuberculous pleural effusion

**DOI:** 10.3389/fimmu.2024.1342641

**Published:** 2024-05-13

**Authors:** Xuan Wang, Chengqing Yang, Chao Quan, Jun Li, Yan Hu, Peng Liu, Lulu Guan, Li Li

**Affiliations:** ^1^ Wuhan Pulmonary Hospital, Wuhan Institute for Tuberculosis Control, Wuhan, Hubei, China; ^2^ Wuhan Center for Clinical Laboratory, Wuhan, Hubei, China

**Keywords:** tuberculosis, tuberculous pleural effusion (TPE), IL-32, immune, IFN-γ

## Abstract

The possible protective effect of interleukin-32 (IL-32) in *Mycobacterium tuberculosis* (*Mtb*) infection has been indicated. However, few studies have been focused on IL-32 in tuberculosis patients. Additionally, the regulation of IL-32 production has rarely been reported. In the present study, the production, regulation, and role of IL-32 in tuberculous pleurisy (TBP) were investigated. We found that the content of IL-32 in tuberculous pleural effusion (TPE) was higher than the level in the malignant pleural effusion and transudative pleural effusion. The level of IL-32 mRNA in pleural fluid mononuclear cells (PFMCs) was higher than that in peripheral blood mononuclear cells (PBMCs) of patients with TBP, and this difference was mainly reflected in the splice variants of IL-32α, IL-32β, and IL-32γ. Compared with the PBMCs, PFMCs featured higher IL-32β/IL-32γ and IL-32α/IL-32γ ratios. In addition, lipopolysaccharide (LPS), Bacillus Calmette-Guérin (BCG), and H37Ra stimulation could induce IL-32 production in the PFMCs. IL-32 production was positively correlated with the TNF-α, IFN‐γ, and IL-1Ra levels in TPE, whereas IFN-γ, but not TNF-α or IL-1Ra, could induce the production of IL-32 in PFMCs. Furthermore, IL-32γ could induce the TNF-α production in PFMCs. Monocytes and macrophages were the main sources of IL-32 in PFMCs. Nevertheless, direct cell–cell contact between lymphocytes and monocytes/macrophages plays an important role in enhancing IL-32 production by monocyte/macrophage cells. Finally, compared with the non-tuberculous pleural effusion, the purified CD4^+^ and CD8^+^ T cells in TPE expressed higher levels of intracellular IL-32. Our results suggested that, as a potential biomarker, IL-32 may play an essential role in the protection against *Mtb* infection in patients with TBP. However, further studies need to be carried out to clarify the functions and mechanisms of the IFN-γ/IL-32/TNF-α axis in patients with TBP.

## Introduction

Tuberculous pleural effusion (TPE), one of the most common forms of extra-pulmonary tuberculosis (TB) (1), is a serious delayed hypersensitivity caused by the collapse of the sub-pleural *Mycobacterium tuberculosis* (*Mtb*) infection focus (2), which usually occurs within 3 to 6 months after the primary infection of *Mtb*. The lower presence of *Mtb* in TPE, along with the limited sensitivity of *Mtb* culture and the technical challenges and invasiveness of thoracoscopy, present obstacles for diagnosing and treating TPE (3). A comprehensive understanding of the host responses to *Mtb* is vitally important for the diagnosis and development of prevention strategies for TPE.

Interleukin-32 (IL-32) was first characterized as a pro-inflammatory cytokine in 2005 (4). Although the IL-32 gene has no sequence homology with other cytokine families, it was called IL-32 because of its powerful pro-inflammatory effects (5). IL-32 mRNA is not only highly expressed in immune cells and tissues but also detected in non-immune tissues and cells (6). More than nine isomers of IL-32 have been found, with the main subtypes being IL-32α, IL-32β, IL-32γ, and IL-32δ (7, 8). Different splice variants play different roles, sometimes even performing opposite functions. IL-32 has recently been found to play a crucial role in the immune response to intracellular pathogenic microorganisms such as HIV and *Mtb*. Stimulating the human PBMCs from healthy donors with heat-killed mycobacterium could elevate the production of IL-32 (9). Additionally, recombinant IL-32 protein can enhance the *in vitro* killing of *Mtb* by macrophages derived from human monocytes (10). Bai X et al. conducted an investigation on the transgenic mice expressing human IL-32γ and found that compared with that in the control group, the growth of *Mtb* in transgenic mice was reduced by 85% (11). In summary, these results showed that IL-32 plays an important role in the immune response to *Mtb*.

Although IL-32 may have a protective role in the anti-*Mtb* response, so far, little is known about the functions of different isomers in the local microenvironment of TPE. In addition, few studies have been performed on patients with tuberculous pleurisy (TBP) to evaluate the function of IL-32. In this study, the expression levels of IL-32 and its isomers in TPE were examined, the reasons why *Mtb* induced IL-32 secretion in TPE were explored, and the immunomodulatory effects of IL-32 during mycobacterial infection were highlighted. Overall, the current research provides a new perspective on the role of IL-32 in the local microenvironment of TPE.

## Materials and methods

### Patients

Active tuberculosis was diagnosed according to i) no previous history of TB; ii) positive acid-fast staining, positive *Mtb* culture, or positive Xpert *Mtb*/RIF test; and iii) no other lung diseases. The exclusion criteria of active TB were i) immunodeficiency, such as HIV, co-existing autoimmune diseases, or long-term steroid use; and ii) a history of anti-tuberculosis treatments, latent tuberculosis infection (LTBI), household contacts of newly diagnosed TB patients with positive interferon-gamma release assay (IGRA), and no evidence of clinical manifestations of active tuberculosis. Other diseases include pulmonary sarcoidosis, lung cancer, and other lung diseases.

A total of 50 patients with tuberculous pleurisy (15 women and 35 men; mean ± SEM age 44.78 ± 2.54 years, range 20 –82 years) were recruited from the Wuhan Pulmonary Hospital, Wuhan, China. A diagnosis of TPE was based on one of the following criteria: i) positive acid-fast staining, ii) pleural fluid or pleural biopsy specimens with growth of *Mtb* culture, iii) *Mtb* DNA testing positive, or iv) with clinical data highly suggestive of tuberculosis and pleural effusion improved after anti-tuberculosis treatment. Patients diagnosed with HIV or autoimmune diseases were excluded from the study. Malignant pleural effusion (MPE) refers to observed malignant cells in pleural effusion or pleural biopsy specimens. This study has been approved by the ethics committees of Wuhan Pulmonary Hospital, approval No. 2023 (43).

### Sample collection and processing

The pleural effusion samples were extracted using standard thoracic puncture techniques, and the peripheral blood samples were drawn simultaneously. Mononuclear cells were separated from the peripheral blood using Ficoll Paque (Tianjin HaoYang, Tianjin, China) gradient centrifugation within 24 hours after sampling, named pleural fluid mononuclear cells (PFMCs) and peripheral blood mononuclear cells (PBMCs), respectively. Then, the cells were re-suspended in the complete Roswell Park Memorial Institute (RPMI) 1640 medium.

### RNA isolation and quantitative real-time PCR

A total of 1 × 10^7^ PFMCs were stimulated with lipopolysaccharide (LPS) at a concentration of 100 ng/mL, Bacillus Calmette-Guérin (BCG) at a ratio of 1:10 (PFMCs : BCG), and H37Ra at a ratio of 1:10 (PFMCs:H37Ra). After incubation at 37°C with 5% CO_2_ for 24 hours, the total RNA was extracted using the TRIzol reagent (Invitrogen, Carlsbad, CA, USA). In addition, freshly separated PBMCs and PFMCs without any cultivation or stimulation were also extracted using the same method. The whole blood RNA was extracted using the QIAamp RNA Blood Mini Kit (QIAGEN, Valencia, CA, USA). Reverse transcription was performed using the kits manufactured by Applied Biosystems, Inc. (Foster City, CA, USA). Then, the PowerUp SYBR Green Master Mix kit was used for the quantitative real-time PCR. GAPDH was used as an internal control gene in the reaction system. The primer sequences are listed in **Table 1**.

**Table 1:** Primer sequences.

Primer name	Sequence
IL-32 forward primer	AGGACGTGGACAGGTGATGTC
IL-32 reverse primer	GTCTCCAGGTAGCCCTCTTTGA
IL-32α forward primer	CGTGGACAGGTGATGTCGAG
IL-32α reverse primer	CTCCGTAGGACTTGTCACAAAA
IL-32β forward primer	AATCAGGACGTGGACAGGTGATGT
IL-32β reverse primer	GTGCCACCAGGTCTGCAGCCG
IL-32γ forward primer	GGTGACTGTCTCAGTGGAGC
IL-32γ reverse primer	CTGTCTCCAGGTAGCCCTCT
IL-32δ forward primer	TCTCTGGTGACATGAAGAAGCT
IL-32δ reverse primer	GCAAAGGTGGTGTCAGTATC
IFN-γ forward primer	TCGGTAACTGACTTGAATGTCCA
IFN-γ reverse primer	TCGCTTCCCTGTTTTAGCTGC
TNF-α forward primer	CCTCTCTCTAATCAGCCCTCTG
TNF-α reverse primer	GAGGACCTGGGAGTAGATGAG
IL-1Ra forward primer	CATTGAGCCTCATGCTCTGTT
IL-1Ra reverse primer	CGCTGTCTGAGCGGATGAA
GAPDH forward primer	GCACCGTCAAGGCTGAGAAC
GAPDH reverse primer	TGGTGAAGACGCCAGTGGA

### ELISAs

According to the protocol of the manufacturer, the concentrations of IL-32, TNF-α, IL-1β, IL-1Ra, IL-17, IL-6 (R&D, Minneapolis, MN, USA), and IFN-γ (BD, San Jose, CA, USA) in the cell culture supernatants, pleural effusion supernatants, or plasma of the participants were measured using the sandwich enzyme-linked immunosorbent assay (ELISA) method (R&D or BD Systems). In a 96-well plate, 200 μL of culture medium containing 5 × 10^5^ PFMCs was added alongside culture medium (control) or different stimuli: LPS (100 ng/mL), BCG (at a ratio of 1:10 with PFMCs), H37Ra (at a ratio of 1:10 with PFMCs), IL-32γ (50 ng/mL), TNF-α (20 ng/mL), IL-1Ra (20 ng/mL), or IFN-γ (20 ng/mL). After the cultivation at 37°C, the supernatant was removed in some experiments. Three freeze–thaw cycles were used to lyse the cells, then the intracellular cytokine concentrations were measured, and the results represented “cell-associated” IL-32 production; intracellular and secreted IL-32 concentrations were determined together, and the results represented “total” IL-32 production (9).

In order to clarify the cellular source of IL-32 production stimulated by *Mtb*, PFMCs were incubated at 37°C for 2 hours. The non-adherent cells that were transferred to another well were mainly lymphocytes, while the majority of adherent cells were monocytes and macrophages. The PFMCs, non-adherent cells, and adherent cells were stimulated with RPMI, LPS (100 ng/mL), BCG (at a ratio of 1:10 with PFMCs), and H37Ra (at a ratio of 1:10 with PFMCs). After 24 hours, the concentrations of “total IL-32” and IFN-γ were measured (9). In co-culture trans-well experiments, the non-adherent cells were added to the top well of the trans-well. Meanwhile, the adherent cells were plated in the lower compartment, stimulated with H37Ra, and co-cultured for 24 hours. Cells and medium were removed from the lower compartment and the top well, and the concentrations of “total IL-32” and IFN-γ were measured.

### Cell isolation and flow cytometry staining and analysis

CD4^+^ T cells and CD8^+^ T cells were isolated by magnetic-activated cell sorting (MACS) using the CD4^+^ T-cell isolation kit and CD8^+^ T-cell isolation kit (Miltenyi Biotec, Bergisch Gladbach, Germany). Human IL-32 APC-conjugated antibody (No. IC30402A) was obtained from R&D Systems (Minneapolis, MN, USA). The CD4^+^ T cells and CD8^+^ T cells from patients with or without TPE were fixed, permeabilized, and stained for intracellular cytokine IL-32 before measurements were taken. Flow cytometry staining was performed using the BD FACS Canto II flow cytometry, and the data were analyzed using FlowJo software.

### Statistical analysis

All analyses were conducted using GraphPad Prism version 9 or SPSS version 23. The normal distribution variables were expressed as mean ± standard error of the mean (SEM), while the non-normal distribution variables were expressed with the interquartile range (IQR). Pearson’s correlation coefficients were computed to assess the relationship between IL-32 and other variables. In addition, Student’s t-test or Mann–Whitney U tests were conducted to examine the differences between every two groups. When the *p*-value was less than 0.05, the difference was considered statistically significant.

## Results

### Demographic, clinical, and laboratory characteristics of the patients with TBP

In total, 50 patients aged 20 to 82 with TBP were enrolled in this study. Among the patients, the male-to-female ratio of cases was 7:3, with an average age of 44.8. The medians of the erythrocyte sedimentation rate (ESR) and C-reactive protein (CRP) in the peripheral blood of the TBP patients were 66 mm/h (IQR 38.5–75.5) and 69.00 (IQR 23.93–82.95) mg/L, respectively. The medians of lactate dehydrogenase (LDH), adenosine deaminase (ADA), and IL-32 in TBP were 403 (IQR 286.5–681) U/L, 41.9 (IQR 30.5–53.35) U/L, and 354.3 (IQR 198.42–529.2) pg/mL, respectively (**Table 2**). The analysis revealed that the level of IL-32 in the TPE was positively correlated with the LDH (Pearson’s *r* = 0.286, *p* < 0.05) and ADA (Pearson’s *r* = 0.391, *p* < 0.01) in TPE. Additionally, the IL-32 level in TPE showed a positive correlation with CRP in the peripheral blood (Pearson’s *r* = 0.293, *p* < 0.05) (**Table 3**).

**Table 2:** Demographic, clinical, and laboratory characteristics of patients with tuberculous pleurisy (n = 50).

Variable	Value
No. of male patients	35/50 (70%)
Age (years)	
Mean ± SEM	44.78 ± 2.54
Range	20–82
Peripheral blood	
ESR (mm/h)	66 (38.5, 75.5)
CRP (mg/L)	69.00 (23.93, 82.95)
Pleural effusion	
TP (g/L)	44.67 ± 1.37
GLU (mmol/L)	5.31 ± 0.37
LDH concn (U/L)	403 (286.5, 681)
ADA concn (U/L)	41.9 (30.50, 53.35)
IL-32 (pg/mL)	354.3 (198.42, 529.2)

ESR, erythrocyte sedimentation rate; CRP, C-reactive protein; ADA, adenosine deaminase; LDH, lactate dehydrogenase; TP, total protein; GLU, glucose.

**Table 3:** Correlation of IL-32 with continuous variables (n = 50).

		Ratio IL-32	*p*-Value
		Pearson’s (*r*)	
Pleural effusion	TP	0.215	0.1337
	GLU	−0.144	0.3171
	LDH	0.286	0.0438*
	ADA	0.391	0.0051**
Peripheral blood	CRP	0.293	0.0389*
	ESR	0.128	0.3757

ESR, erythrocyte sedimentation rate; CRP, C-reactive protein; ADA, adenosine deaminase; LDH, Lactate dehydrogenase; TP, total protein; GLU, glucose.

*p < 0.05; **p < 0.01.

### IL-32 mRNA expressions in the whole blood from active pulmonary tuberculosis, latent pulmonary tuberculosis, healthy individuals, and other diseases

**Figure 1:**
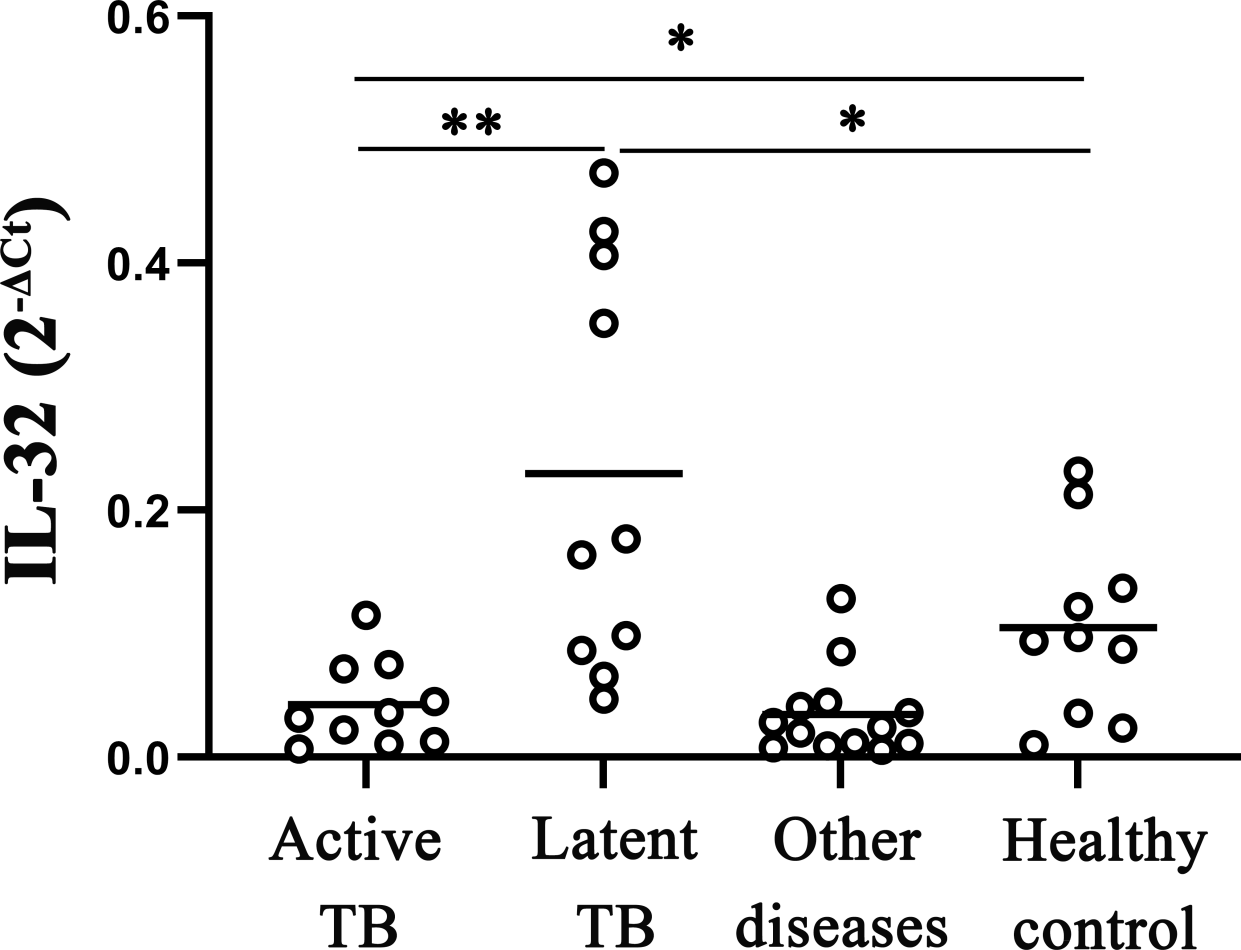
IL‐32 mRNA expression measured by fluorescence quantitative PCR in whole blood. The levels of IL-32 mRNA in active pulmonary tuberculosis, latent pulmonary tuberculosis, healthy individuals, and other lung diseases (including pneumonia, malignant tumors, and various other lung infections). *p < 0.05; **p < 0.01.

The study analyzed the levels of IL-32 mRNA in active pulmonary tuberculosis, latent pulmonary tuberculosis, healthy individuals, and other diseases. Results showed that IL-32 mRNA expression was higher in latent tuberculosis patients compared to that in active pulmonary tuberculosis and healthy individuals. The IL-32 mRNA in active tuberculosis patients was lower than that in healthy individuals (all *p* < 0.05) (**Figure 1**). In addition, compared with the other groups, in the group of other diseases (including pneumonia, malignant tumors, and various other lung infections, which need to make a differential diagnosis with TB), the expression of IL-32 mRNA was the lowest (*p* < 0.05).

### Concentration of IL-32 in TPE, MPE, and transudative pleural effusion

**Figure 2:**
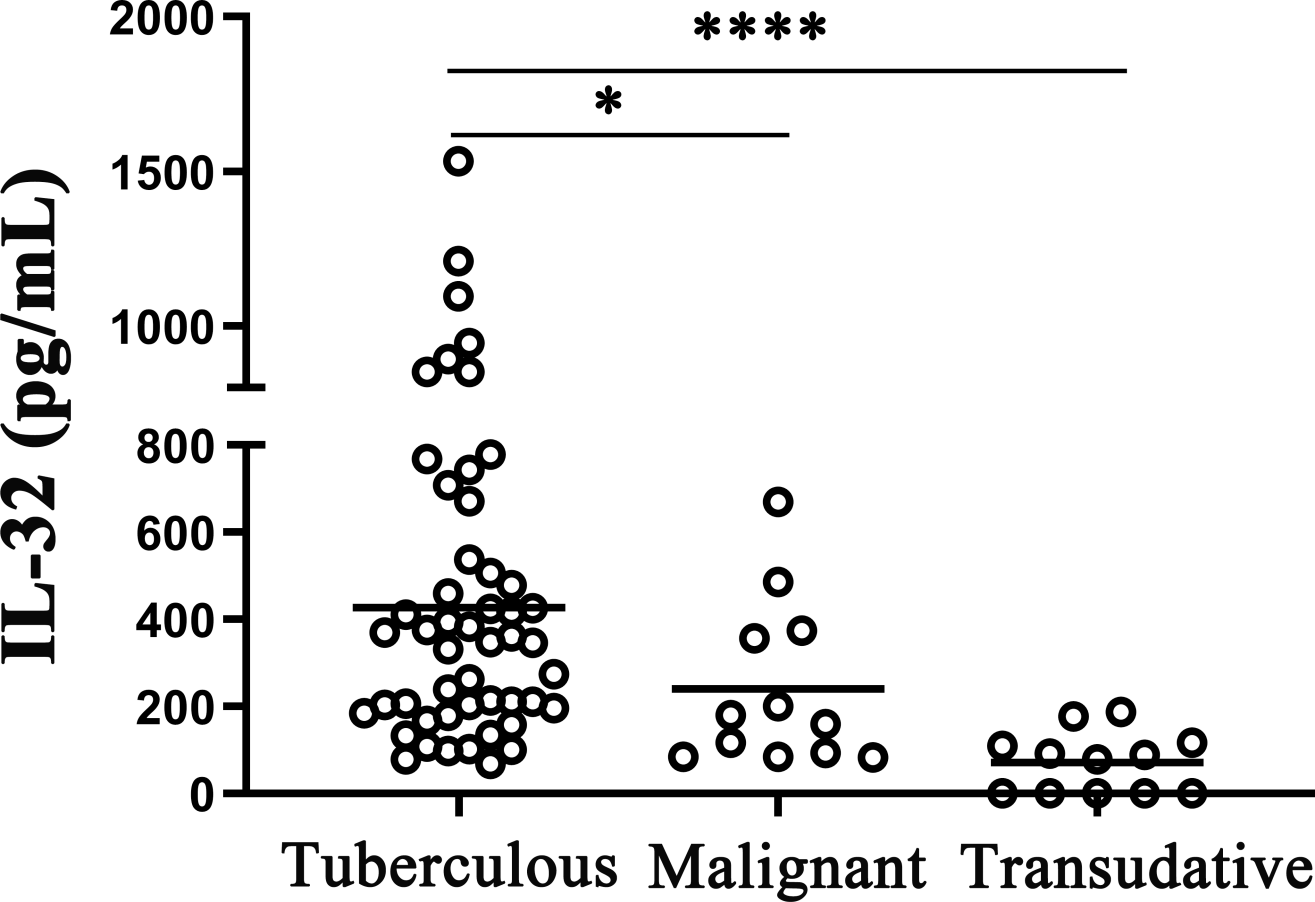
IL-32 levels in different pleural effusions. The concentrations of IL-32 in tuberculosis pleural effusion (n = 50), malignant pleural effusion (n = 12), and transudative pleural effusion (history of heart disease) (n = 12) were measured by ELISA. *p < 0.05; ****p < 0.0001.

Furthermore, the IL-32 expressions in the TPE, MPE, and transudative pleural effusion were checked. The average concentrations of IL-32 in TPE were significantly higher than in MPE and transudative pleural effusion (such as the history of heart disease) (all *p* < 0.05). There were no differences between MPE and transudative pleural effusion (*p* > 0.05) (**Figure 2**).

### IL-32 in TPE and PBMCs in patients with TBP

**Figure 3:**
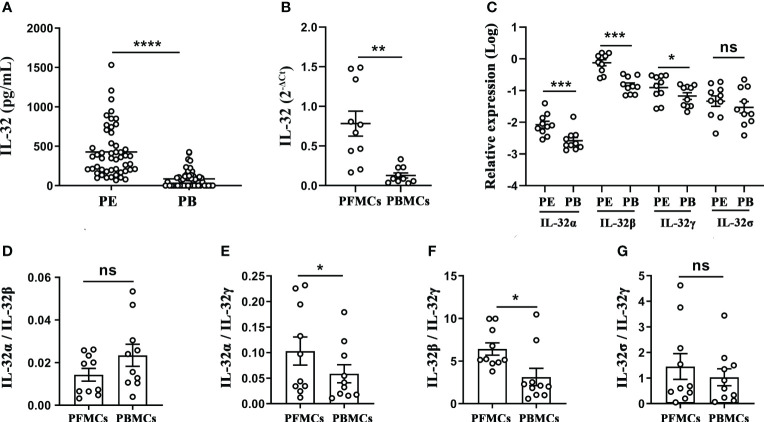
Analysis of IL-32 and its isoforms in pleural effusion and peripheral blood of patients with tuberculous pleurisy. **(A)** IL-32 concentration in pleural effusion and plasma was measured by ELISA (n = 50). **(B, C)** The levels of IL-32, IL-32α, IL-32β, IL-32γ, and IL-32δ in mononuclear cells from TPE and paired peripheral blood were measured using quantitative PCR and normalized against the housekeeping gene GAPDH, and the relative expression was calculated as 2^(−ΔΔCt)^ (mean fold change ± SEM, n = 10). **(D–G)** The ratios of IL-32α/IL-32β, IL-32α/IL-32γ, IL-32β/IL-32γ, and IL-32δ/IL-32γ in PFMCs and PBMCs were compared (mean ± SEM, n = 10). *p < 0.05; **p < 0.01; ***p < 0.001; ****p < 0.0001. TPE, tuberculous pleural effusion; PFMCs, pleural fluid mononuclear cells; PBMCs, peripheral blood mononuclear cells.

The concentrations of IL-32 in the TPE and paired peripheral blood were measured, and the results indicated that the concentration of IL-32 in the TPE was significantly higher than that in the paired peripheral blood (*p* < 0.0001, **Figure 3A**), and IL-32 mRNA in the PFMCs was significantly higher than that in the PBMCs (*p* < 0.01, **Figure 3B**). Furthermore, IL-32α, IL-32β, and IL-32γ mRNA levels in the PFMCs were higher than those in the paired PBMCs (all *p* < 0.05), while no difference in IL-32δ was observed between the two groups (*p* > 0.05) (**Figure 3C**). The ratios of IL-32α/IL-32γ (**Figure 3E**) and IL-32β/IL-32γ (**Figure 3F**) in the PFMCs were higher than those in the PBMCs (*p* < 0.05). However, IL-32α/IL-32β (**Figure 3D**) and IL-32δ/IL-32γ (**Figure 3G**) did not differ between the PFMCs and PBMCs, indicating that the alternative splicing of IL-32γ into IL-32α and IL-32β was significantly enhanced in the PFMCs compared with the paired PBMCs. These data suggest that different IL-32 subtypes may have different roles and regulated patterns in the local TPE and peripheral blood immune inflammatory response in patients with TBP.

### H37Ra and BCG induced production of IL-32 in PFMCs

**Figure 4:**
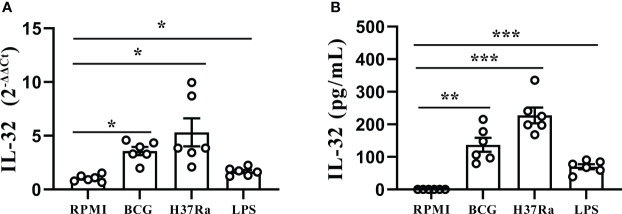
Stimulation of IL-32 by *Mtb* stimulus. **(A)** PFMCs isolated from fresh TPE were stimulated with RPMI, LPS, BCG, and H37Ra for 24 hours. IL-32 mRNA was measured by quantitative PCR and normalized against the housekeeping gene GAPDH, and the expression was calculated as 2^(−ΔΔCt)^ (mean fold change ± SEM, n = 6). **(B)** PFMCs were stimulated with RPMI, LPS, BCG, or H37Ra; Triton X-100 (0.5%) was added 24 hours later; IL-32 concentration was measured by ELISA. Data are presented as means ± SEM (n = 6). *p < 0.05; **p < 0.01; ***p < 0.001. PFMCs, pleural fluid mononuclear cells; TPE, tuberculous pleural effusion; RPMI, Roswell Park Memorial Institute; LPS, lipopolysaccharide; BCG, Bacillus Calmette-Guérin.

To further investigate the inductive effect of different *Mtb* stimuli on IL-32 production, freshly obtained PFMCs were stimulated with LPS, BCG, and H37Ra, and then the gene expressions and concentrations of IL-32 were determined. As shown in **Figure 4A**, the IL-32 mRNA expression increased after the addition of LPS, BCG, and H37Ra (all *p* < 0.05). Simultaneously, the production of IL-32 induced by the various stimuli was detected. Higher levels of total IL-32 production were observed in response to stimulation with H37Ra (227.4 ± 23.9 pg/mL, *p* < 0.001) and BCG (151.8 ± 22.6 pg/mL, *p* < 0.01), while LPS (71.6 ± 8.7 pg/mL, *p* < 0.001) induced a more moderate increase in IL-32 production (**Figure 4B**). Additionally, no IL-32 was detected in the culture supernatant of the PFMC or RPMI group, suggesting that the IL-32 levels in the culture supernatant of the PFMC and RPMI groups were even lower than the lowest value limit of the IL-32 cytokine detection kit.

### IL-32 correlated positively with the IFN-γ, TNF-α, and IL-1Ra levels in TPE

**Figure 5:**
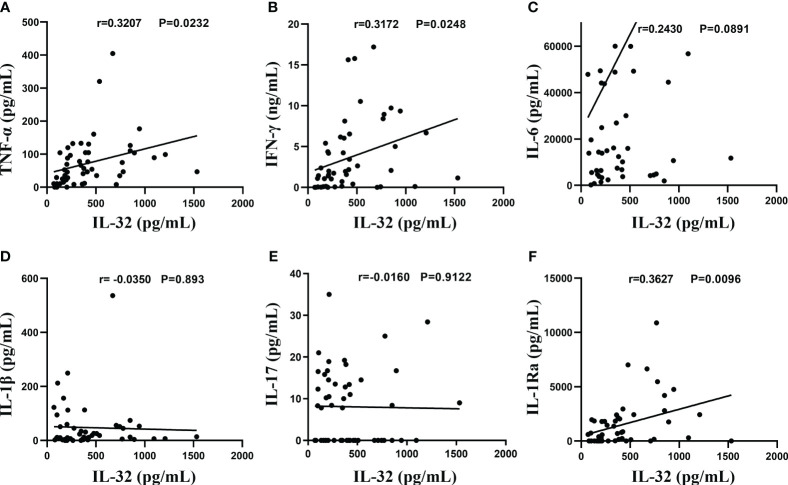
Association between IL-32 and other cytokines. **(A–F)** The concentrations of IL-32, TNF-α, IFN-γ, IL-6, IL-1β, IL-17, and IL-1Ra in tuberculosis pleural effusion were measured by ELISA. Pearson’s correlation coefficients were calculated for the relationship between IL-32 and cytokines (n = 50).

As shown in **Figure 5**, in the TPE, the levels of TNF-α (**Figure 5A**), IFN-γ (**Figure 5B**), and IL-1Ra (**Figure 5F**) showed a significant correlation with IL-32 levels (*r* = 0.3207, *p* < 0.05; *r* = 0.3172, *p* < 0.05; *r* = 0.3627, *p* < 0.01), while no significant correlation was observed for other cytokines such as IL-6 (**Figure 5C**), IL-1β (**Figure 5D**), and IL-17 (all *p* > 0.05) (**Figure 5E**).

### IFN-γ induced production of IL-32 in PFMCs

**Figure 6:**
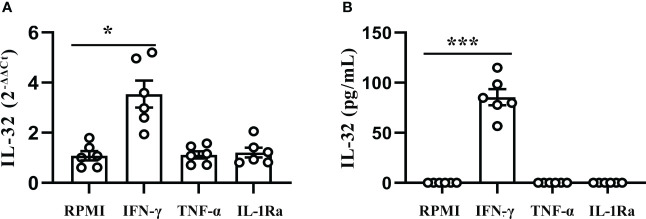
IFN-γ induced production of IL-32 in PFMCs. **(A)** PFMCs isolated from fresh TPE were treated with IFN-γ (20 ng/mL), IL-1Ra (20 ng/mL), and TNF-α (20 ng/mL) for 24 hours; mRNA expression of the IL-32 was measured by quantitative PCR (mean fold change ± SEM, n = 6). **(B)** PFMCs isolated from fresh TPE were stimulated with IFN-γ (20 ng/mL), IL-1Ra (20 ng/mL), and TNF-α (20 ng/mL) for 24 hours. Total IL-32 production was measured by ELISA 24 hours later in cells lysed with Triton X-100 (mean fold change ± SEM, n = 6). *p < 0.01; ***p < 0.001. PFMCs, pleural fluid mononuclear cells; TPE, tuberculous pleural effusion.

To further evaluate the correlation between IL-32 and the above cytokines, PFMCs were stimulated with human recombinant protein IFN-γ, TNF-α, and IL-1Ra. As shown in **Figure 6A**, PFMCs stimulated with IFN-γ protein induced a 3.3-fold change in IL-32 mRNA expression (*p* < 0.05). However, TNF-α and IL-1Ra failed to stimulate the expression of IL-32 mRNA in PFMCs. In addition, IFN-γ enhanced the production of IL-32 significantly (*p* < 0.001, **Figure 6B**), while IL-32 was not detected in the culture medium control group, TNF-α group, and IL-1Ra group.

### IL-32 induced production of TNF-α in PFMCs

**Figure 7:**
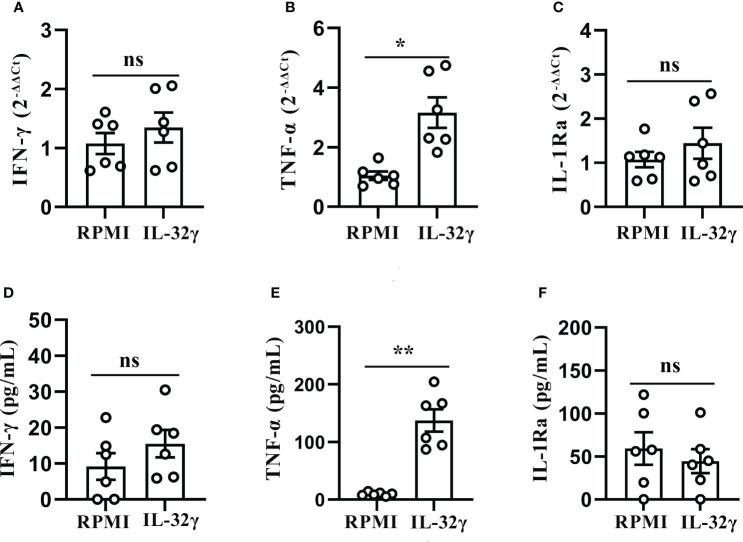
IL-32 induced production of TNF-α in PFMCs. **(A–C)** PFMCs isolated from tuberculous pleurisy patients were stimulated with IL-32 (50 ng/mL), IFN-γ, and TNF-α; IL-1Ra mRNA was measured by quantitative PCR and normalized against the housekeeping gene GAPDH, and the expression was calculated as 2^(−ΔΔCt)^ (mean fold change ± SEM, n = 6). **(D–F)** PFMCs isolated from tuberculous pleurisy patients were stimulated with IL-32 (50 ng/mL); concentrations of IFN‐γ were measured by ELISA after 2 days. TNF-α and IL-1Ra concentrations were measured by ELISA 24 hours later. *p < 0.05; **p < 0.01. PFMCs, pleural fluid mononuclear cells.

Considering IL-32γ was the most active isoform (12, 13), PFMCs were treated with human reorganizing IL-32γ protein, and the results showed that the IL-32γ treatment positively induced the TNF-α mRNA expression (**Figure 7B**) and secretion (**Figure 7E**), and compared with the RPMI control group, the difference was statistically significant (*p* < 0.05). However, there was no difference in the expression and production of other cytokines between IL-32γ-treated PFMCs and untreated cells (**Figures 7A, C, D, F**).

### Monocytes and macrophages were the main sources of IL-32 in PFMCs

**Figure 8:**
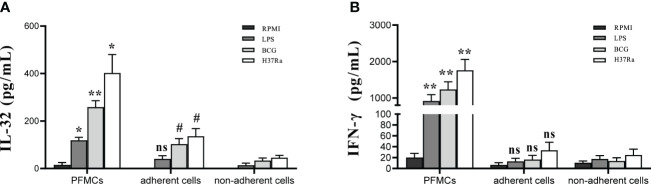
Cell source of IL-32 Production in PFMCs. PFMCs were incubated at 37°C for 2 hours, and non-adherent cells were transferred to another well. The non-adherent cells transferred to another well were mainly lymphocytes. PFMCs, adherent cells, or non-adherent cells were stimulated with RPMI, H37Ra, BCG, or LPS. After 24 hours, **(A)** IL-32 and **(B)** IFN-γ concentrations were measured. *PFMCs *vs.* adherent cells, ^#^non-adherent cells *vs.* adherent cells. ns, no statistical difference; * and ^#^ *p* < 0.05, **p < 0.001. PFMCs, pleural fluid mononuclear cells; RPMI, Roswell Park Memorial Institute; LPS, lipopolysaccharide; BCG, Bacillus Calmette-Guérin.

PFMCs were separated into non-adherent cells and adherent cells. PFMCs, adherent cells, and non-adherent cells were stimulated with RPMI, H37Ra, BCG, and LPS for 24 hours. Compared to that in non-adherent lymphocytes, the concentration of “total IL-32” in stimulated adherent cells was significantly higher (*p* < 0.05) (**Figure 8A**). Compared with that in PFMCs, the total production of IL-32 by separated adherent cells and non-adherent cells was reduced (all *p* < 0.05) (**Figure 8A**). The concentration of IFN-γ in the stimulated PFMCs was significantly higher compared to either stimulated adherent or non-adherent lymphocytes (*p* < 0.01) (**Figure 8B**). In addition, no significant difference was detected as to the production of IFN-γ between stimulated adherent cells and non-adherent cells (*p* > 0.05) (**Figure 8B**).

To further verify whether the increase of IL-32 in adherent cells is related to direct cell–cell contact with non-adherent cells, trans-well co-culture assays were conducted. PFMCs, adherent cells, non-adherent cells, and co-cultured cells were separately stimulated with H37Ra for 24 hours. The results revealed higher IL-32 production in PFMCs compared to adherent cells and co-cultured cells (*p* < 0.05) (**Supplementary Figure 1A**). No significant difference was found as to IL-32 expression between adherent cells and co-cultured cells (*p* > 0.05) (**Supplementary Figure 1A**). Moreover, IFN-γ production by PFMCs was higher than that of adherent cells and co-cultured cells (*p* < 0.01), while no difference was observed between adherent cells and co-cultured cells in terms of IFN-γ levels (*p* > 0.05) (**Supplementary Figure 1B**).

### Intracellular IL-32 in purified CD4^+^ T and CD8^+^ T cells from TPE and non-TPE

**Figure 9:**
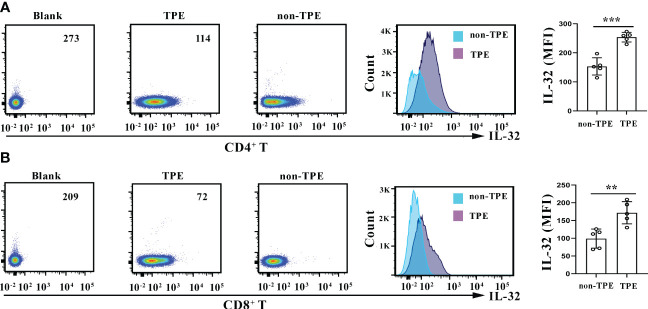
IL-32 staining in CD4^+^ and CD8^+^ T cells. **(A)** Density plots and overlay histogram showing IL-32 expression in CD4^+^ T cells in TPE and non-TPE. **(B)** Density plots and overlay histogram showing IL-32 expression in CD8^+^ T cells in TPE and non-TPE. MFI, mean fluorescence intensity; TPE, tuberculous pleural effusion. Data are presented as means ± SEM (n = 6). **p < 0.01; ***p < 0.001.

Research has shown an increase in IL-32 expression in macrophages in TPE compared to non-TPE (14). Therefore, the expression of IL-32 was further compared within T cells between TPE and non-TPE. CD4^+^ and CD8^+^ T cells were purified from TPE and non-TPE, respectively. The flow cytometry results revealed that the expression of IL-32 was enhanced in the CD4^+^ T cells purified from TPE compared with the CD4^+^ T cells purified from non-TPE (*p* < 0.001) (**Figure 9A**). Similarly, IL-32 was also enhanced in the CD8^+^ T cells purified from TPE compared with that from non-TPE (*p* < 0.01) (**Figure 9B**).

## Discussion

Thoracic effusion detection is considered a minimally invasive method for distinguishing between TPE and non-TPE. However, to date, accurate and stable biomarkers that can reliably confirm TPE in pleural effusion have not been reported, thereby posing a huge challenge to the diagnosis and treatment of TPE (15). Previous studies have shown that IL-32 has a protective effect on *Mtb* (16). Herein, we observed that the level of total IL-32 mRNA in the whole blood of patients with pulmonary tuberculosis decreased compared with that in patients with latent tuberculosis infection and healthy controls, indicating its possible role in congenital prevention of *Mtb* infection. We found that IL-32 was positively correlated with LDH and ADA in TPE. ADA has been used in diagnosing TPE (17), while LDH can be used to distinguish certain malignant tumors (18). Both ADA and LDH can be used for differential diagnosis of TPE. These studies indicated that IL-32 in pleural effusion may be a potential biomarker to distinguish TPE from other pleural effusions and the combination of clinical indicators and IL-32 may have significant diagnostic value for the properties of pleural effusion. So far, studies on IL-32 in TBP have been rarely reported as well, and various TPE patients were thus hereby analyzed to further explore these hypotheses in primary cells. In this study, we explored the relationship between IL-32 and anti-*Mtb* infection in the TBP.

The local IL-32 content was higher than the peripheral IL-32 content, indicating the differences in host local and peripheral immune responses. TPE had a higher level of IL-32 compared with the MPE and transudative pleural effusion, indicating the unique role of IL-32 in the process of occurrence and development of TB. In addition, we observed that H37Ra and BCG induced IL-32 production in PFMCs; this *in vitro* experiment validates the viewpoint of *in vivo* experiments. IL-32 has been proven to be a pro-inflammatory cytokine that can induce other cytokines involved in inflammation (19). Some studies have demonstrated that CRP increased in tuberculosis patients, and these levels declined with the progress of treatment (20, 21). Herein, IL-32 was found to be significantly correlated with clinical inflammation indicator CRP, further validating the pro-inflammatory effect of IL-32. Different splice variants of pro-inflammatory cytokine IL-32 have been found in various tissues, but their differences in biological function remain unknown. IL-32γ is considered the longest and most active subtype of IL-32 and can be spliced into the lower active subtypes IL-32β and IL-32α (22). In this study, the IL-32β/IL-32γ and IL-32α/IL-32γ ratios in PFMCs were found to feature a selective splicing enhancement compared with PBMCs. Previous studies on colitis and rheumatoid arthritis patients have shown that IL-32γ selective splicing to IL-32β plays a role in self-restraint of uncontrolled inflammation, which can be a salvage mechanism to reduce inflammation (23, 24). Therefore, it was proposed that the increase in splicing events of IL-32β and IL-32α in TPE may contribute to the self-restraint of TPE inflammation and may be related to prognosis. Based on previously published studies, these data further confirmed that IL-32 and different IL-32 isoforms had very different roles and regulatory patterns in the tuberculosis process, forging a strong basis for measuring different IL-32 isoforms in future laboratory and clinical studies.

IFN-γ has been repeatedly proven to be crucial in host defense TB (25). IFN-γ pathway defects are accompanied by increased susceptibility to low-virulence mycobacterium infections. Based on IL-32 being positively correlated to IFN‐γ and that human recombinant IFN‐γ protein could stimulate the production and secretion of IL-32 in PFMCs, it was hereby speculated that IFN-γ could regulate the expression of IL-32, and it appeared that IFN-γ was a highly selective IL-32-inducing cytokine in TPE of patients with TBP. Previous studies have confirmed that silencing the endogenous IL-32 in THP-1 macrophages can significantly reduce the TNF-α, IL-1β, and IL-8 production and simultaneously increase the burden of *Mtb* on infected macrophages (16). Recombinant human IL-32γ protein induces a large amount of TNF production in RAW 264.7 macrophages (26). Similarly, individuals with TBP and IL-32γ-treated PFMCs showed increased TNF-α levels compared to untreated cells. Our findings may be one of the reasons explaining the role of IL-32 in controlling the immune response of tuberculosis, indicating that IFN-γ/IL-32/TNF-α signaling axis may play an important role in the pathogenesis of tuberculosis.

In the present study, we found that monocytes and macrophages were the main sources of IL-32 in PFMCs, whereas lymphocytes were not the major cell source of IL-32, and their presence increased the production of IL-32 by adherent cells in PFMCs significantly. IL-32 production by PFMCs was significantly higher than that in co-cultured adherent and non-adherent cells, indicating that direct cell–cell contact plays an important role in enhancing IL-32 production by monocytes/macrophages. Moreover, no significant difference was detected as to IL-32 expression by adherent cells and co-cultured cells. Therefore, the addition of non-adherent cells into the upper well had little effect on the production of IL-32 expression by adherent cells in the lower compartment, probably due to a lack of IFN-γ production by T cells, which mostly depended on direct cell–cell contact between T cells and monocytes/macrophages following antigen stimulation. Additionally, we found that the expression of IL-32 by CD4^+^ T and CD8^+^ T cells in TPE was higher than that in non-TPE. Effective T cells play a crucial role in anti-tuberculosis immunity (27), and previous studies have reported an association between IL-32 and T cells. For example, IL-32 is linked to the balance of Th1/Th17 cytokines in the peripheral blood of patients with pulmonary tuberculosis (8) and high levels of IFN-γ and TNF-α expression in T cells in lung tissue of IL-32γ-transgenic mice infected with *Mtb* (11). However, the role of IL-32 in T cell- or monocyte/macrophage-mediated immunity in TPE remains unclear, necessitating further comprehensive investigation in the future.

In healthy individuals, steady-state mRNA levels of IL-32 are present in newly obtained blood PBMCs (28). Herein, it was also found that steady-state mRNA levels were present in un-stimulated PFMCs and PBMCs in patients with TBP, and PFMCs featured a higher IL-32 mRNA expression than PBMCs. Similar to other cytokines, such as IL-1β, they all lacked classic signaling peptides (29) and thus are not transported through the classical endoplasmic reticulum and Golgi-mediated pathway (30). In addition, IL-32 is a cell-related cytokine, so measurable IL-32 was found present in the lysates of these cells. To this end, in stimulated pleural effusion mononuclear cells, there was no detectable IL-32 in the supernatant, and it was not surprising that the cell lysate contained almost all measurable IL-32.

The regulatory role of IL-32 and its isomers in the immune system is complex and requires further elucidation of the molecular mechanisms involved. Understanding the production of IL-32 is crucial for enhancing our comprehension of the immune system and exploring new diagnostic and treatment strategies for conditions like TPE and tuberculosis. Therefore, additional research is imperative to uncover the mechanisms of IL-32 and its splice variants in tuberculosis.

## Conclusion

Some cytokines and growth factors lack clear signaling peptides and cannot be secreted yet play a significant role in the pathogenesis of diseases. In summary, the results of this study provide some important insights into the biology of IL-32. First, monocytes and macrophages were the main cell source of IL-32 in PFMCs. Although lymphocytes were not the major cell source of IL-32, their direct contact with non-adherent monocytes/macrophages plays an important role in enhancing IL-32 production by monocyte/macrophage cells. Second, mycobacterium species induce IL-32 production by CD4^+^ T and CD8^+^ T cells in human TPE. Third, the content of IL-32 detected locally in TPE is higher than that in other pleural effusions, which may be attributed to the stimulation of local *Mtb* in TPE. Moreover, IL-32 is a cell-related pro-inflammatory cytokine closely related to clinical inflammatory indicators. Finally, the ability of *Mtb* to stimulate IL-32, i.e., a key cytokine for host defense against these microorganisms, is related to its mediated IFN-γ secretion, and its anti-*Mtb* effect is at least partially related to IFN-γ and TNF-α secretion. Overall, the above research is expected to provide new ideas for the immune diagnosis, disease prognosis, and disease treatment of TPE patients. In the future, further investigation will be carried out on the role and molecular regulatory mechanisms of the IFN-γ/IL-32/TNF-α signaling axis in patients with TBP.

## Data availability statement

The raw data supporting the conclusions of this article will be made available by the authors, without undue reservation.

## Ethics statement

The studies involving humans were approved by Ethics committees of Wuhan Pulmonary Hospital. The studies were conducted in accordance with the local legislation and institutional requirements. The participants provided their written informed consent to participate in this study.

## Author contributions

XW: Data curation, Formal analysis, Funding acquisition, Methodology, Project administration, Writing – original draft. CY: Data curation, Formal analysis, Investigation, Writing – review & editing. CQ: Investigation, Writing – review & editing. JL: Data curation, Investigation, Writing – review & editing. YH: Data curation, Investigation, Writing – review & editing. PL: Data curation, Writing – review & editing. LG: Data curation, Writing – review & editing. LL: Investigation, Methodology, Project administration, Supervision, Writing – review & editing, Conceptualization.

## Supplementary material

Supplementary Figure 1 IL-32 and IFN-γ production by trans-well co-cultured cells. PFMCs, adherent cells, non-adherent cells, and co-cultured cells were stimulated with H37Ra, respectively. After 24 hours, concentrations of IL-32 **(A)** and IFN-γ **(B)** were measured. ns, no statistical difference, *, P<0.05, **, P<0.001.
