# Supplementary figures and images for: The regulation and potential role of interleukin-32 in tuberculous pleural effusion
Source: Front Immunol. 2024 May 13;15:1342641. doi: 10.3389/fimmu.2024.1342641 (PMC11128554; doi:10.3389/fimmu.2024.1342641)

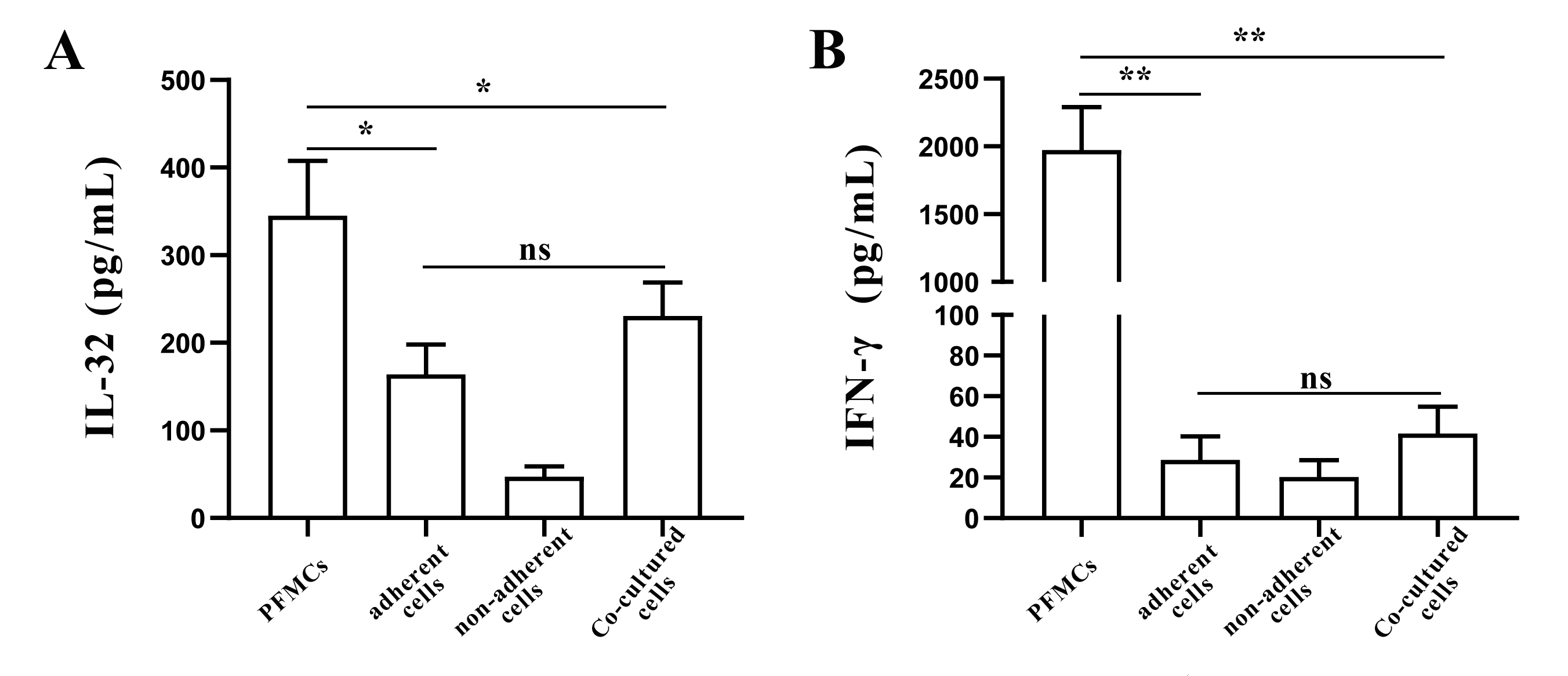

Supplement: Supplementary Figure 1 — IL-32 and IFN-γ production by trans-well co-cultured cells. PFMCs, adherent cells, non-adherent cells, and co-cultured cells were stimulated with H37Ra, respectively. After 24 hours, concentrations of IL-32 (A) and IFN-γ (B) were measured. ns, no statistical difference, *, P<0.05, **, P<0.001. [file Image_1.tif]
